# Epidemiology and seasonality of respiratory syncytial virus among hospitalized pediatric patients with lower respiratory tract infections in Negeri Sembilan, Malaysia (2017–2024)

**DOI:** 10.3389/fpubh.2026.1947126

**Published:** 2026-10-26

**Authors:** Nuur Hafizah Md Iderus, Sarbhan Singh, David Chun-Ern Ng, Sumarni Mohd Ghazali, Nur’ain Mohd Ghazali, Mei Cheng Lim, Nur Ar Rabiah Ahmad, Qistina Ruslan, Chuin-Hen Liew, Marlindawati Mohd Ali, Nor Zanariah Zainol Abidin, Nurul Hafizah Mohd Yusoff, Balvinder Singh Gill, Mohd Azahadi Omar, Lonny Chen Rong Qi Ahmad

**Affiliations:** 1 Biomedical Epidemiology Unit, Special Resource Centre, Institute for Medical Research, National Institutes of Health, Ministry of Health, Shah Alam, Selangor, Malaysia; 2 Hospital Tuanku Jaafar, Ministry of Health, Seremban, Negeri Sembilan, Malaysia; 3 Department of Pediatrics, Faculty of Medicine and Health Sciences, Universiti Putra Malaysia, Serdang, Selangor, Malaysia; 4 Department of Pediatrics, Hospital Sultan Abdul Aziz Shah, Universiti Putra Malaysia, Serdang, Selangor, Malaysia; 5 National Public Health Laboratory, Ministry of Health, Sungai Buloh, Selangor, Malaysia; 6 Special Resource Centre, Institute for Medical Research, National Institutes of Health, Ministry of Health, Shah Alam, Selangor, Malaysia

**Keywords:** ARIMAX, correlation, pediatric, respiratory syncytial virus, seasonality

## Abstract

**Background:**

Respiratory Syncytial Virus (RSV) remains a significant public health concern globally, with distinct seasonal patterns in temperate regions, while tropical settings experience RSV year-round with seasonal fluctuations. This study investigated seasonality and exogenous factors influencing RSV cases among hospitalized children under 12 years in Negeri Sembilan, Malaysia.

**Methods:**

Weekly laboratory-confirmed RSV-positive cases among hospitalized pediatric patients presenting with acute lower respiratory tract infections (LRTIs) across 417 continuous WHO epidemiological weeks (EpiWeek 1, 2017 to EpiWeek 52, 2024) were analyzed using decomposition and autocorrelation functions. Relationships between RSV and co-circulating viruses, meteorological variables, and holidays were assessed via cross-correlation and Autoregressive Integrated Moving Average with Exogenous variables (ARIMAX) modeling.

**Results:**

A total of 6,545 (14.29%) samples were RSV-positive from a total of 45,817 tested. Historical peaks (July–September) were disrupted during 2020–2023 but re-established in 2024. The ARIMAX (2,0,4) (1,0,0)_52_ model revealed that RSV dynamics were driven primarily by internal autoregressive patterns rather than meteorological factors. Major public holidays exhibited a significant dual effect, with an immediate decrease in reported cases (*p* = 0.012) followed by a significant surge (*p* = 0.014). Influenza activity was a significant predictor with a one-week lag (*p* = 0.012).

**Conclusion:**

The pandemic temporarily shifted RSV seasonality, which has since normalized. The associations with holiday periods and influenza activity suggests that behavioral factors and viral interference may influence RSV circulation. These findings emphasize the need for dynamic surveillance to optimize the timing of future RSV immunizations in Malaysia.

## 1 Introduction

Respiratory syncytial virus (RSV) remains a leading cause of acute lower respiratory tract infections globally, particularly in infants and children, where it is estimated to cause 33 million cases, 3.6 million hospital admissions, and over 100,000 deaths annually (1). The disease also presents a significant public health burden in the Asia Pacific region, with respiratory diseases being the second leading cause of hospitalization in Malaysia (2). RSV positivity rates tend to vary widely across geographical regions due to variation in testing protocols and patient populations. In Malaysia, studies have reported RSV positivity rates among children ranging from 15 to 16% from 2015 to 2021 (3, 4). This consistent positivity rate over time results in RSV being a principal driver of hospitalization for acute respiratory tract infections among children in Malaysia. Such a persistent burden underscores the need for continuous surveillance and the development of targeted management strategies, particularly as new preventive interventions become available.

Management of RSV infections remains largely supportive, as no effective RSV-specific antivirals are currently available. Prevention is therefore crucial to reducing disease burden and improving clinical outcomes, especially in young children. Historically, RSV prevention has relied on palivizumab, a monoclonal antibody (mAb) administered to high-risk groups such as premature infants or those with bronchopulmonary dysplasia or congenital heart disease. However, its high cost and narrow eligibility criteria have limited its impact on overall disease burden, which is predominantly seen in otherwise healthy children. Recent advances in RSV prevention, including maternal vaccination and long-acting mAb such as Nirsevimab and Clesrovimab, offer promising population-wide protection for all infants. However, the optimal timing of these preventive interventions depends heavily on understanding RSV epidemics, which are influenced by several complex interrelated factors affecting RSV transmission, such as seasonal trends, meteorological factors, co-circulating respiratory viruses, and human behavioral patterns.

RSV exhibits distinct seasonal trends influenced by geographic and climatic factors. In temperate regions, the virus demonstrates well-defined seasonality, typically peaking during winter months, which facilitates the precise timing of preventive measures (1). Conversely, RSV circulates year-round in tropical countries with inconsistent fluctuations. Within Southeast Asia, peak activity is not uniform; while Thailand (5) and Malaysia (4) share a similar peak period between July and December, neighboring Singapore (June–August) (6) and Indonesia (February–May) (7) experience distinct timings. Notably, the COVID-19 pandemic caused a marked reduction or absence of these typical seasonal peaks (4, 8, 9) and post-pandemic seasonality patterns have yet to be fully re-established.

Beyond seasonality, transmission is influenced by meteorological factors such as rainfall, temperature, wind speed, and humidity, though literature remains inconsistent (10, 11). While temperate transmission increases during colder months tropical surges are often tied to the rainy season (12). In Malaysia, RSV was previously reported to correlate positively with rainfall but negatively with humidity and temperature (13); however, a recent study found a positive correlation with temperature (14). Similar conflicts exist regionally; for instance, Thailand reported positive correlations with rainfall and humidity but a negative correlation with temperature (15).

In addition to meteorological factors, RSV circulation tends to occur as part of a complex respiratory viral ecosystem rather than in isolation. Hence, the dynamics of RSV transmission are influenced by several co-circulating viral infections, such as Adenovirus, Human Metapneumovirus, Influenza, and Parainfluenza viruses (11, 16, 17). This interplay can manifest as co-infection, viral interference, or facilitation, and understanding these interactions is crucial for comprehensive epidemiological analysis of RSV transmission (18, 19).

Public holidays, which are characterized by the closure of public institutions and increased social interactions, travel, and family gatherings, are another important factor that influences human behavior, which has been reported to affect the transmission dynamics of RSV, as observed in studies from Singapore, Sweden, and China (6, 20, 21). This influence is typically attributed to two factors: an increase in viral transmission due to heightened social mixing and a temporary decrease in case reporting due to clinic and laboratory closures.

To effectively evaluate these multi-faceted relationships, an analytical framework capable of handling non-stationary, auto correlated, and seasonally patterned surveillance data is required. Traditional linear regression models fail to account for serial temporal dependencies, while standard ARIMA models cannot incorporate exogenous drivers. Autoregressive Integrated Moving Average with Exogenous Variables (ARIMAX) modeling provides an ideal framework by simultaneously capturing underlying autoregressive structures, seasonal cycles (s = 52), and the immediate or delayed impacts of external meteorological, holiday, and viral co-circulation factors.

While RSV seasonality is well-characterized in temperate regions, its transmission dynamics in tropical settings remain less clearly defined and appear more variable, especially following the disruption of seasonal patterns during the COVID-19 pandemic in many countries. Hence, the current study aims to address these critical gaps by comprehensively investigating RSV transmission among children hospitalized from 2017 to 2024 by (a) determine cumulative and weekly RSV case counts and viral positivity rates; (b) examine seasonal RSV patterns; (c) evaluate time-lagged cross-correlations between RSV cases, co-circulating respiratory viruses, meteorological parameters, and public holiday periods; and (d) identify independent predictors of weekly RSV dynamics using multivariable time-series modeling Findings from this study will provide valuable insights into RSV epidemiology in a tropical context, enhancing our understanding of its transmission patterns and their potential drivers.

## 2 Methods

### 2.1 Study design and setting

A retrospective observational study design was undertaken in Negeri Sembilan, a state located in the western part of Peninsular Malaysia spanning an area of approximately 6,654km^2^ with a population exceeding 1.2 million residents and a pediatric population aged ≤ 12 years of approximately 250,000. The study population comprised hospitalized children aged 12 years and below across all seven Ministry of Health (MOH) government hospitals in the state. These hospitals were in Seremban (Hospital Tuanku Ja’afar;1,143 beds), Kuala Pilah (Hospital Tuanku Ampuan Najihah; 314 beds), Port Dickson (116 beds), Jempol (108 beds), Tampin (108 beds), Jelebu (76 beds) and Rembau (76 beds; Supplementary Figure 1). Hospital bed capacity, population demographic data, and geographic shapefiles were obtained from the Ministry of Health (MOH) Malaysia, Department of Statistics Malaysia (DOSM), and the Database of Global Administrative Areas (GADM), respectively.

The study period spanned 417 continuous epidemiological weeks from January 1, 2017 through December 28, 2024 (EpiWeek 1, 2017 to EpiWeek 52, 2024), encompassing seven standard 52-week calendar years and the 53-week WHO calendar year in 2020. The study focused on the pediatric age group as they represent the highest burden of RSV disease and hospitalizations. In addition, this timeframe was selected as it adequately covers the pre-COVID-19 period (2017–2019), the acute pandemic disruption period (2020–2021) and the post-COVID-19 period (2022–2024), allowing for the full observation of the pandemic’s impact on RSV seasonality. In addition to the primary state-level analysis, a secondary sub-analysis was conducted across three representative districts representing urban (Seremban), coastal (Port Dickson), and inland (Kuala Pilah) demographic zones to evaluate sub-regional spatial consistency.

### 2.2 Data source

- RSV and Co-circulating Viruses

Weekly aggregated case counts for RSV and the co-circulating viruses were sourced from the respective hospital database. Respiratory sampling, using nasopharyngeal aspirates, was performed on hospitalized children with lower respiratory tract infections (LRTI). These samples were tested using direct fluorescent antibody (DFA) assays. DFA detects active viral antigens directly within respiratory epithelial cells, confirming acute infection rather than circulating serum antibodies. The respiratory virus panel included RSV, Human Metapneumovirus (HMPV), Influenza A/B (INFV), adenovirus (ADEV), and parainfluenza virus 1/2/3 (PARAV). Specimens demonstrating multi-viral antigen positivity were recorded under each respective viral pathogen for descriptive prevalence, while all RSV-positive detections were included in the primary RSV time-series analysis. Diagnostic testing across all seven public hospitals in Negeri Sembilan was standardized using multiplex DFA. DFA was selected over RT-PCR due to resource and cost constraints given the high volume of pediatric admissions.

- Meteorological Data

Meteorological variables were sourced from the Malaysian Meteorological Department for Negeri Sembilan. Weekly aggregation was performed for all variables: total cumulative rainfall (in millimeters, mm), mean temperature (in degrees Celsius, °C), and mean wind speed (in meters per second, m/s). Relative humidity was recorded as the mean weekly percentage of relative humidity (%).

- Holiday Data

Public holidays were sourced from the Malaysian Employers Federation.^1^ School holiday data for the same period were obtained from the Ministry of Education.^2^ For this study, school holiday periods were defined to include the mid-term and year-end term breaks and extended breaks during major festivals such as Hari Raya Aidilfitri, Chinese New Year, and Deepavali. The holiday regressor (HOL) was constructed as a continuous numeric variable representing the cumulative count of national and state public holidays and school holiday days occurring within each week (ranging from 0 to 7 days per week).

### 2.3 Data analysis

The complete surveillance dataset spanning a 417-week time series was analyzed descriptively to determine case counts and positivity rates for RSV and co-circulating respiratory viruses. To preserve temporal continuity and maintain the strict 52-week annual seasonal periodicity (s = 52), weeks corresponding to the COVID-19 pandemic disruption (2020–2021) were retained in their original chronological positions. Structural baseline shifts during lockdown weeks were controlled for in multivariable modeling using a formal step-intervention regressor (D_pandemic_) (22). The analytical framework proceeded through three sequential stages described below.

- Descriptive Analysis and Positivity Rates
  Annual and overall cumulative diagnostic positivity rates (%) were calculated for RSV and co-circulating respiratory viruses as positive antigen detections divided by the total number of diagnostic tests executed. Longitudinal trajectories were plotted weekly to examine baseline dynamics and post-lockdown resurgence trends.
- Seasonal analysis

For seasonality analysis, the initial data exploration involved decomposing the raw RSV time series into its components (trend, cycle, seasonality, and random irregular events) using classical additive decomposition methods (s = 52). Seasonality was further analyzed using the autocorrelation function (ACF) and partial autocorrelation function (PACF) plots, and visualized through seasonal subseries plots and district-level peak synchrony heatmaps. Visual evaluation of weekly RSV heatmaps across three representative districts (Seremban, Port Dickson, and Kuala Pilah) using a standardized shared color scale demonstrated synchronized temporal timing of annual seasonal peaks.

- Correlation and cross-correlation analysis

Spearman’s rank correlation (*ρ*) was used to assess monotonic relationships between RSV cases and continuous meteorological factors, holidays, and co-circulating viruses. Normality pre-tests were omitted as non-parametric rank correlations do not assume Gaussian distributions. Correlations were classified as weak (|*ρ*| < 0.30), moderate (0.30 ≤ |*ρ|* ≤ 0.70), or strong (|*ρ|* > 0.70). To prevent spurious cross-correlations caused by shared underlying autocorrelation and 52-week seasonality, both the target RSV series and candidate exogenous series were pre-whitened using a parsimonious baseline filter. Pre-whitened cross-correlation function (CCF) analysis was conducted across Lags 0 to 4 weeks to screen candidate exogenous variables for significant bivariate lead–lag associations (*p* < 0.05) while filtering out spurious temporal trends.

- Autoregressive Integrated Moving Average with Exogenous variables (ARIMAX) analysis

ARIMAX models were developed to assess the combined influence of co-circulating viruses, meteorological factors, holidays, and pandemic disruptions on weekly RSV cases. To account for non-pharmaceutical interventions (NPIs) and lockdown disruptions without introducing data gaps or truncating the continuous time series, a Box-Tiao step-indicator intervention regressor was incorporated into the exogenous matrix (xreg) (22). This regressor was parameterized as a binary step variable (D_pandemic_ = 1 for all weeks in 2020–2021; D_pandemic_ = 0 otherwise).

Seasonal ARIMAX (p,d,q)(P, D, Q)_52_ modeling was conducted following the Box-Jenkins methodology to evaluate the independent associations of co-circulating viruses, meteorological parameters, holidays, and pandemic disruptions on weekly pediatric RSV incidence. Stationarity for all continuous candidate series (RSV cases, co-circulating viral counts, and meteorological parameters) was evaluated using the Augmented Dickey-Fuller (ADF) test (*p* < 0.05), establishing that no first-order differencing (d = 0) was required before multivariable modeling. Automated grid searches (auto.arima()) based on Maximum Likelihood Estimation (MLE) were initially used to explore baseline structures, with candidate specifications evaluated via Akaike Information Criterion (AIC), Corrected AIC (AICc), and Bayesian Information Criterion (BIC). Candidate exogenous variables identified during CCF screening, alongside variables driven by prior epidemiological hypotheses specifically capturing post-holiday transmission rebound (HOL_lag1_), were incorporated into the multivariable regression matrix (xreg), with final regressor retention determined by partial coefficient significance (*p* < 0.05) and overall model information criteria minimization (AIC/BIC). The final multivariable specification was explicitly parameterized to optimize residual white-noise properties.

Because constructing 1-week lagged predictor variables (HOL_lag1_ and INFV_lag1_) requires previous-week values (t − 1), the first two boundary weeks served as initialization baselines. Omitting these initial boundary rows yielded an effective sample size of 415 continuous weekly estimation observations (EpiWeek 3, 2017 through EpiWeek 52, 2024) for final model parameter fitting.

Model adequacy was validated via residual diagnostics, with the Ljung-Box test at lag 52 indicating white-noise residuals (*p* > 0.05) after adjusting for model degrees of freedom. All analyses were performed in R software (v4.3.2) using the forecast, tseries, lmtest, MMWRweek, ggplot2 and other standard functions.

## 3 Results

### 3.1 Descriptive analysis

Annual data revealed that RSV consistently maintained the highest case numbers each year compared to other co-circulating viruses throughout the study period. However, during the COVID-19 years, RSV case numbers were lower compared to the other years. Weekly RSV case and positivity rate were presented in Figure 1. Across the total of 45,817 diagnostic tests performed from 2017 to 2024, RSV exhibited the highest cumulative diagnostic positivity rate (*n* = 6,545;14.29%), followed by INFV (*n* = 1,770;3.86%), PARAV (*n* = 1,208;2.64%), HMPV (*n* = 1,143;2.49%), and ADEV (*n* = 718;1.57%; Table 1). Weekly RSV case counts were plotted alongside co-circulating viral, meteorological parameters, and holiday days across the complete 417-week surveillance window (Supplementary Figure 2).

**Figure 1:**
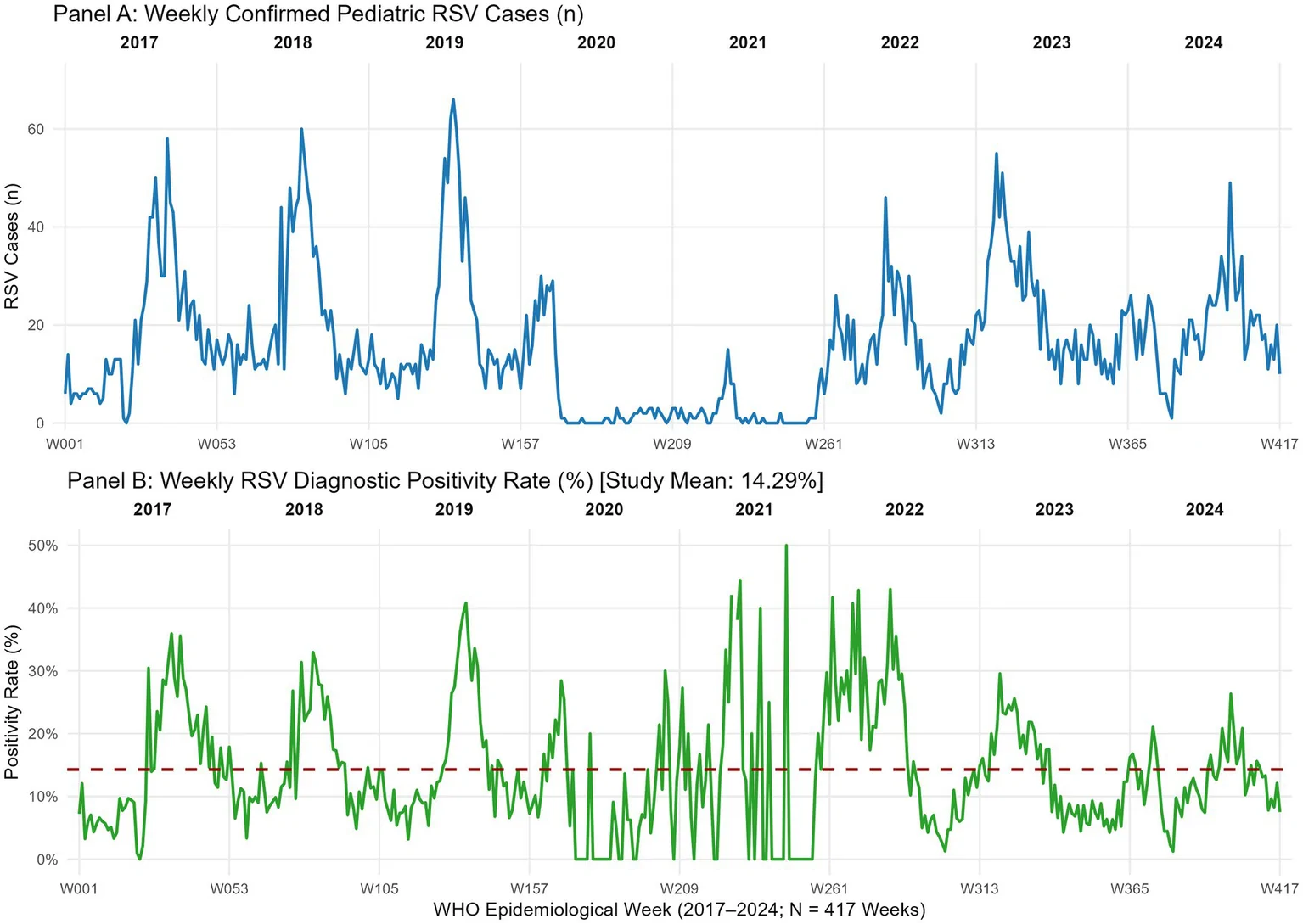
Weekly pediatric RSV surveillance dynamics across 417 WHO epidemiological weeks (EpiWeek 1, 2017 to EpiWeek 52, 2024). **(A)** Weekly RSV cases (*n* = 6,545). **(B)** Weekly positivity rate (%) plotted against total weekly laboratory testing (*N* = 45,817 total diagnostic tests).

**Table 1:** Annual diagnostic testing denominators, RSV case counts and co-circulating viral antigen detections in Negeri Sembilan (2017–2024).

Year	Total epidemiological week	Total Tests	RSV (n)	Influenza (n)	Parainfluenza (n)	Human Metapneumovirus (n)	Adenovirus (n)
2017	52	6,176	945	169	85	87	77
2018	52	7,352	1,122	222	247	164	132
2019	52	6,515	1,049	268	210	127	58
2020	53	2,407	311	202	66	37	39
2021	52	551	108	1	7	1	5
2022	52	5,759	842	166	186	351	126
2023	52	9,263	1,176	426	263	169	229
2024	52	7,794	992	316	144	207	52
Total	417	45,817	6,545	1,770	1,208	1,143	718

### 3.2 Seasonal analysis

Seasonal analysis of weekly RSV cases from 2017 to 2024 revealed a consistent annual seasonal pattern during pre-pandemic years (2017–2019), with case volumes increasing notably between EpiWeeks 25 and 38 (July to September) and frequently exceeding 40 cases per week. This established seasonal structure was severely disrupted during the COVID-19 pandemic period (2020–2021), during which RSV transmission was markedly suppressed or virtually absent due to COVID-19 NPIs (Figure 2).

**Figure 2:**
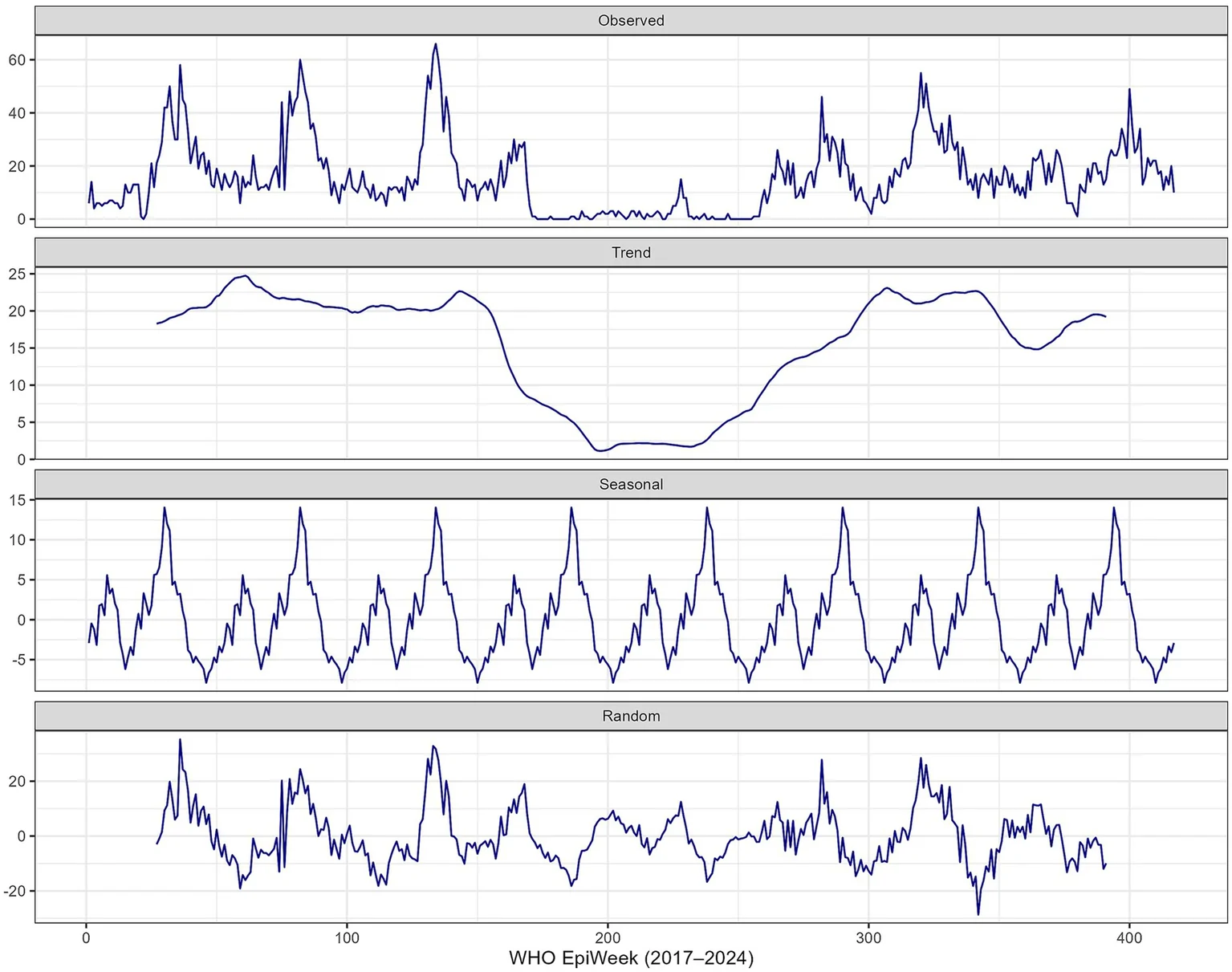
Classical additive seasonal decomposition (*s* = 52) of the continuous 417-week RSV time series. Panel 1: Observed weekly case counts (*n* = 6,545). Panel 2: Long-term non-linear trend component. Panel 3: Recurring 52-week annual seasonal cycle (*s* = 52). Panel 4: Irregular residual component.

Following the relaxation of mobility restrictions in late 2021, post-lockdown resurgence exhibited altered temporal timing: in 2022, the seasonal transmission peak appeared earlier, between EpiWeeks 20 and 24 (May to June). A distinct seasonal shift occurred in 2023, with peak RSV activity concentrated in the first quarter of the year (EpiWeeks 4 to 12; January to March). By 2024, however, RSV seasonality re-established its pre-pandemic temporal alignment, once again peaking during the mid-year July–September period.

Classical additive seasonal decomposition of the weekly RSV series revealed a dominant, recurring 52-week seasonal component (s = 52) operating beneath pandemic-induced baseline shifts (Figure 2). Autocorrelation function (ACF) and partial autocorrelation function (PACF) diagnostic plots of the decomposed series demonstrated prominent spikes at seasonal lag depths (Lag_52_), indicating strong 52-week seasonal dependence and justifying the inclusion of seasonal ARIMA operators (P, D, Q)_52_ in subsequent ARIMAX modeling. Furthermore, cross-district visual and numerical evaluation demonstrated high spatial synchrony of these annual peaks across urban (Seremban), coastal (Port Dickson), and inland (Kuala Pilah) demographic zones, justifying the aggregation of statewide pediatric data for time-series modeling (Supplementary Figure 3).

### 3.3 Correlation and cross-correlation

Spearman correlation screening revealed moderate positive associations between weekly pediatric RSV incidence and co-circulating respiratory viruses, including INFV (ρ = 0.490), HMPV (ρ = 0.457), ADEV (ρ = 0.413), and PARAV (ρ = 0.413). A moderate inverse correlation was observed for the COVID-19 pandemic regressor (ρ = −0.615), reflecting systemic case suppression. Conversely, environmental parameters including mean temperature (ρ = 0.024), relative humidity (ρ = 0.010), total rainfall (ρ = −0.123), and wind speed (ρ = 0.112) demonstrated weak associations (Table 2).

**Table 2:** Monotonic correlation coefficients and pre-whitened cross-correlation function (CCF) values across Lags 0 to 4 weeks for viral, meteorological, and holiday exogenous regressors.

Predictor	Variable code	Spearman (ρ)	Lag0	Lag1	Lag2	Lag3	Lag4
Pandemic Regressor	Dpandemic	−0.615***	−0.012	−0.031	−0.032	−0.038	−0.025
Holidays	HOL	−0.021	−0.086*	0.033	0.083	0.128*	0.014
Influenza	INFV	0.49***	0.122**	0.001	−0.054	−0.024	−0.022
Adenovirus	ADEV	0.413***	0.093	−0.008	−0.026	−0.02	−0.02
Parainfluenza	PARAV	0.413***	0.083	0.013	−0.018	0.013	−0.018
Human Metapneumovirus	HMPV	0.457***	0.057	0	−0.002	0.062	−0.015
Mean Temperature (°C)	TEM	0.024	0.011	0.013	−0.058	0.021	0.036
Relative Humidity (%)	HUM	0.01	−0.04	−0.036	0.071	−0.054	−0.083
Total Rainfall (mm)	RAIN	−0.123*	−0.03	−0.066	0.028	−0.065	−0.043
Mean Wind Speed (m/s)	WIND	0.112*	0.066	0.029	−0.013	0.008	0.008

Statistical significance levels: *p < 0.05, **p < 0.01, ***p < 0.001. Pre-whitening filters applied prior to CCF calculation to eliminate serial autocorrelation artifacts.

Pre-whitened cross-correlation function (CCF) screening was conducted across Lags 0 to 4 weeks to eliminate spurious correlations arising from shared temporal trends and autocorrelation (Table 2). Following pre-whitening, INFV was the only co-pathogen to maintain a statistically significant independent cross-correlation, exhibiting an immediate positive spike at Lag 0 (CCF = 0.122, *p* < 0.01). Conversely, ADEV, PARAV, HMPV, and all meteorological parameters (temperature, humidity, rainfall, wind speed) demonstrated non-significant pre-whitened cross-correlations (*p* > 0.05). ADEV was further excluded during preliminary variable selection to prevent multicollinearity. Public and school holidays (HOL) demonstrated a distinct dual-phase structure: an immediate negative association during active holiday weeks (Lag_0_: CCF = −0.086, *p* < 0.05), reflecting operational healthcare-seeking delays, followed by a delayed positive rebound in subsequent weeks (Lag_3_: CCF = 0.128, *p* < 0.05), capturing post-holiday transmission expansion. Consequently, D_pandemic_(Lag_0_), HOL (Lags_0_, Lag_1_, and Lag_3_), and INFV (Lag_0_) were retained to populate the candidate multivariable ARIMAX exogenous regression matrix (Table 2).

### 3.4 Multivariable ARIMAX model analysis

Building upon the seasonal decomposition (Figure 2) and pre-whitened cross-correlation lag structures (Table 2), level-stationarity for all continuous candidate series was verified via the ADF test (*p* < 0.05). Initial univariate baseline modeling using auto.arima() identified an ARIMA (2,0,2; 1,0,0)_52_ topology. Upon introducing selected exogenous regressors, the non-seasonal moving-average component was expanded to q = 4, yielding the final multivariable ARIMAX (2,0,4; 1,0,0)_52_ specification.

Controlling for baseline pandemic suppression and autoregressive memory, overall RSV transmission dynamics were significantly driven by specific exogenous viral co-circulations and holiday dynamics rather than local meteorological factors. Major public and school holidays exerted a significant dual-phase transmission pattern: an immediate concurrent reduction in reported RSV cases during active holiday weeks (HOL_lag0_: *β* = −0.319, SE = 0.127, *p* = 0.012), followed by a significant delayed transmission surge one-week post-holiday (HOL_lag1_: *β* = 0.312, SE = 0.126, *p* = 0.014). Among co-circulating respiratory pathogens, influenza activity was a statistically significant positive predictor of RSV incidence at a 1-week lag (INFV_lag1_: *β* = 0.200, SE = 0.080, *p* = 0.012). In contrast, environmental meteorological parameters (mean temperature, relative humidity, and total rainfall) did not retain statistically significant independent associations in the final multivariable matrix.

Visual validation of model trajectories demonstrated high predictive fidelity between observed weekly pediatric RSV cases and ARIMAX model predictions across all 415 effective estimation weeks (EpiWeek 3, 2017 through EpiWeek 52, 2024; Figure 3). The final multivariable model demonstrated strong goodness-of-fit, achieving an AIC of 2702.46, AICc = 2703.36, BIC of 2754.89, and a log-likelihood of −1338.23 (residual variance σ^2^ = 36.82). The model significantly outperformed candidate baseline univariate specifications (ΔAIC > 12.2). Formal white-noise evaluation via the Ljung-Box test at lag 52 justified that residual errors were homoscedastic and serially independent (χ^2^ = 55.11, df = 45, *p* = 0.144; Supplementary Figure 4).

**Figure 3:**
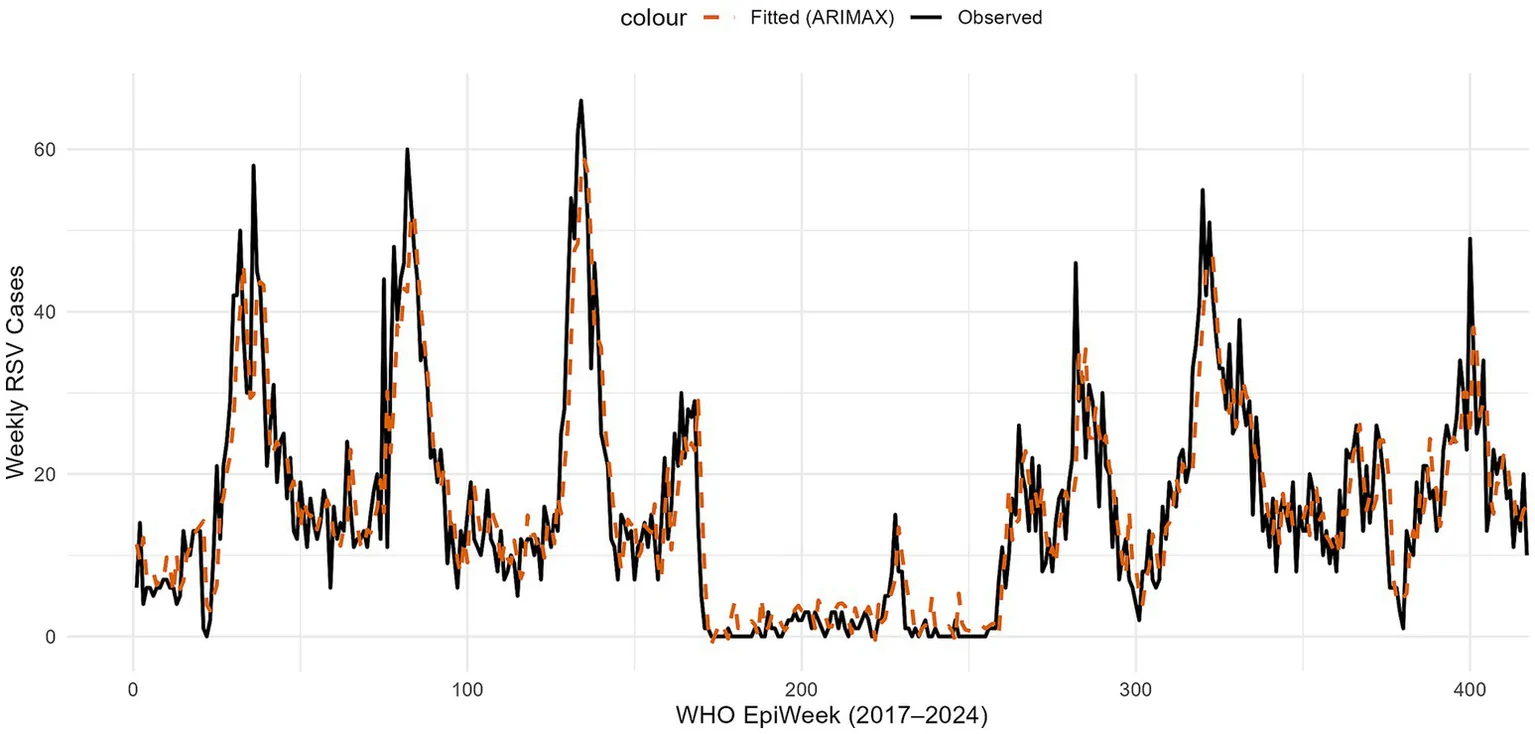
Continuous time-series overlay of observed weekly RSV case counts (black line) versus fitted values from the multivariable ARIMAX (2,0,4) (1,0,0)_52_ model (dashed line) across 415 effective estimation weeks (2017–2024).

## 4 Discussion

This study provides a comprehensive temporal evaluation of pediatric RSV case dynamics across Negeri Sembilan, Malaysia, leveraging an 8-year surveillance matrix spanning 417 continuous WHO epidemiological weeks (2017–2024). By incorporating a step-intervention dummy variable (D_pandemic_) within a multivariable ARIMAX framework, our model effectively captured long-term epidemiological drivers while controlling for baseline suppression events during the COVID-19 pandemic. This analytical workflow allowed precise characterization of recurring seasonal patterns, pre-whitened lead–lag relationships with exogenous factors, and the identification of robust transmission drivers within an equatorial tropical setting.

Beyond laboratory surveillance, clinical management of severe pediatric LRTIs increasingly leverages non-invasive bedside modalities. Promising diagnostic imaging tools, particularly lung point-of-care ultrasound and advanced non-invasive chest assessment techniques, have demonstrated substantial utility in differentiating viral pneumonia, evaluating consolidation severity, and monitoring disease progression without radiation exposure in pediatric populations (23, 24). Integrating continuous molecular/DFA surveillance with real-time non-invasive clinical imaging protocols represents an important avenue for refining inpatient triage and severity tracking during peak seasonal RSV surges.

RSV was the most prevalent respiratory pathogen in this study (14.29% positivity), aligning with local data reporting 15.1% (4) and underscoring RSV as a leading cause of pediatric acute lower respiratory infections. High cumulative prevalence reflects the virus’s significant transmissibility and lack of sustained post-infection immunity in children, contrasting with the episodic nature of influenza and lower prevalence of HMPV, adenovirus, and parainfluenza. Pre-pandemic RSV activity consistently peaked from July to September; however, these trends were suppressed during 2020–2021 due to COVID-19 NPIs (4, 25). Pre-pandemic seasonality did not immediately resume upon easing NPIs in 2022, only fully re-establishing by 2024; a delayed recovery driven by “immunity debt,” altered health-seeking behaviors, and staggered school reopenings (26, 27).

This temporary suppression and post-pandemic resurgence aligns with broader global epidemiological shifts (28). More broadly, our baseline findings align with global systematic evidence characterizing tropical RSV dynamics (29, 30). A comprehensive systematic analysis across China highlighted a distinct latitudinal gradient, demonstrating that warm tropical and subtropical regions experience extended viral persistence (>6 months) or biannual peaks, a pattern consistent with our finding, unlike the short winter outbreaks typical of temperate zones (28). Global multi-country syntheses similarly show that tropical settings frequently experience extended transmission windows and higher year-on-year peak variability compared to temperate zones (29, 30). Within this regional context, the re-establishment of a predictable mid-year peak (July–September) in Negeri Sembilan indicates that despite continuous year-round background activity, tropical RSV transmission in Peninsular Malaysia remains anchored by strong annual periodicity.

The pre-whitened cross-correlation analysis provided critical insights into the lagged relationships governing pediatric RSV dynamics prior to multivariable model fitting. A weak negative correlation with rainfall was initially observed, aligning with previous studies in Malaysia and other tropical settings where heavy precipitation reduces social mixing and indoor gathering density (14, 31). However, after pre-whitening both series using a baseline ARIMA structure to remove autocorrelation and shared 52-week seasonality, climate variables, including temperature, relative humidity, and rainfall, did not remain statistically significant (32, 33). In contrast, structural non-climatic factors and co-circulating viral dynamics indicated significant time-lagged relationships with RSV residuals (33, 34). Public and school holidays exhibited a dual-phase effect, showing an immediate suppression of cases due to temporary school closures, followed by a positive rebound the following week that reflects post-holiday transmission resumption. Among co-circulating pathogens, Influenza at a one-week lag was uniquely retained, highlighting shared seasonal transmission windows and overlapping pediatric population susceptibility (35). Furthermore, NPIs during the COVID-19 pandemic exerted a strong baseline step-suppression effect. These pre-whitened relationships indicated that public movement patterns, pandemic interventions, and specific viral co-circulators, rather than direct meteorological parameters, served as the primary exogenous drivers of pediatric RSV transmission in this equatorial setting.

Multivariable ARIMAX (2,0,4) (1,0,0) ₅₂ modeling demonstrated that pediatric RSV transmission in Negeri Sembilan is governed by strong endogenous autoregressive memory alongside structured external drivers, maintaining a robust 52-week annual seasonality. Public and school holidays continued to show a significant influence, exhibiting a distinct dual-phase pattern: an immediate negative effect at Lag 0 followed by a positive rebound at Lag 1. This dual pattern likely reflects reduced healthcare utilization and testing during holiday closures, followed by a surge in delayed clinical reporting or increased transmission from heightened social mixing shortly after the break (20, 35). Additionally, a significant positive association with Influenza at a 1-week lag in the overall model suggests a consistent interplay and shared transmission windows between these two major respiratory viruses. However, because population-level time-series modeling captures ecological co-occurrence, these associations generate hypotheses regarding shared environmental drivers rather than establishing direct biological viral interference at the cellular level (25–27). Crucially, accounting for the COVID-19 pandemic indicator as a baseline step regressor controlled for pandemic-era suppression, demonstrating that while NPIs temporarily altered incidence, underlying transmission dynamics resumed once public movement normalized. These findings collectively emphasize that the factors influencing RSV are not static, underscoring the need for continued, region-specific studies to inform effective public health strategies.

The findings from this study carry important clinical and public health implications. The return of a predictable seasonal peak following the COVID-19 pandemic reinforces the importance of sustained surveillance and timely clinical preparedness during peak transmission periods, especially from July to September. Clinicians should anticipate surges in children’s LRTI admissions during these months and ensure adequate resource allocation, such as hospital beds, trained personnel, and ventilators. Although this study demonstrated a seasonal peak of RSV in the state, RSV activity was observed throughout the year, with a substantial burden outside the peak periods. This highlights the limitations of a seasonal immunoprophylaxis strategy in tropical settings. Given the persistent year-round circulation and vulnerability of young infants, a year-round prophylaxis strategy may be more appropriate to ensure broader protection, especially to those born outside the seasonal peak (Table 3).

**Table 3:** Multivariable ARIMAX (2,0,4) (1,0,0)_52_ model parameter estimates.

Parameter	Estimate	Std_Error	z_value	p_value
ar1	0.074	0.232	0.317	0.751
ar2	0.704	0.207	3.397	0.001^***^
ma1	0.508	0.236	2.156	0.031*
ma2	−0.119	0.097	−1.230	0.219
ma3	0.199	0.061	3.280	0.001***
ma4	0.121	0.056	2.169	0.030*
sar1	0.046	0.058	0.788	0.430
intercept	16.623	2.493	6.668	0.000***
Dpandemic	−7.852	3.536	−2.220	0.026*
HOL_lag0	−0.319	0.127	−2.521	0.012*
HOL_lag1	0.312	0.126	2.469	0.014*
INFV_lag1	0.200	0.080	2.513	0.012*

Model fit statistics: AIC = 2702.46, AICc = 2703.36 BIC = 2754.89, Log-Likelihood = −1338.23, σ^2^ = 36.82. Ljung-Box test at lag 52: χ^2^ = 55.11, df = 45, *p* = 0.144. *p < 0.05, **p < 0.01, ***p < 0.001.

Despite these valuable findings, this study has several limitations. First, case capture was restricted to hospitalized pediatric patients with acute lower respiratory tract infections, omitting milder outpatient presentations. Pathogen surveillance across the seven public hospitals also relied on multiplex DFA due to cost constraints that made statewide RT-PCR prohibitive; however, because testing protocols remained strictly uniform across all 417 weeks, DFA’s lower analytical sensitivity acts as a constant baseline offset that preserves the validity of seasonality trends, peak timing, and ARIMAX lag relationships. Additionally, patient-level clinical data (e.g., symptom severity, oxygen requirements, length of stay) were not recorded, precluding clinical severity subgroup analyses. Second, state-level aggregation of meteorological data across varied microclimates (coastal vs. inland), alongside the lack of age-stratified incidence rates, population immunity metrics, and granular mobility data, may introduce spatial and demographic exposure misclassification. Third, controlling for pandemic-era disruptions via a binary step regressor did not capture micro-level operational shifts; future research should evaluate deep learning architectures (e.g., Deep Neural Network and Stacked Long Short-Term Memory) (36–38) and digital symptom query trends (e.g., Google Trends, eVision, and ARGO frameworks) (39–41) for non-linear feature extraction and real-time epidemic nowcasting during structural shocks. Finally, findings from a single Malaysian state may not fully generalize to regions with differing sociodemographic or climatic profiles. Future research should integrate age-stratified surveillance, localized mobility tracking, and digital query tracking to further elucidate tropical RSV dynamics.

## 5 Conclusion

Longitudinal surveillance across an 8-year dataset provides empirical evidence that pediatric RSV in Malaysia follows a predictable mid-year seasonal peak alongside year-round background transmission. An ARIMAX time-series model demonstrated that historical seasonal patterns fully returned after temporary disruptions during the COVID-19 pandemic. Key drivers of transmission included co-circulating Influenza and a dual-phase holiday effect, where active holidays briefly delayed hospital presentations before triggering post-holiday case rebounds. These findings underscore the importance of dynamic surveillance strategies, particularly during holiday periods, in effectively managing RSV outbreaks in Malaysia.

## Data Availability

The datasets presented in this article are not readily available because the data used in this study belong to the Ministry of Health Malaysia. Access to the datasets requires official permission from the Director General of Health, Malaysia. Requests to access the datasets should be directed to Ministry of Health Malaysia.

## Footnotes

1
www.mef.org.my

2
www.moe.gov.my
