# Supplementary material for: Epidemiology and seasonality of respiratory syncytial virus among hospitalized pediatric patients with lower respiratory tract infections in Negeri Sembilan, Malaysia (2017–2024)
Source: Front Public Health. 2026 Oct 26;14:1947126. doi: 10.3389/fpubh.2026.1947126 (PMC13761330; doi:10.3389/fpubh.2026.1947126)
Supplement: Supplementary file 1 [file Data_Sheet_1.docx]

Supplementary Figure 1. Spatial distribution of public Ministry of Health (MOH) hospital centers, district population, and bed capacity across Negeri Sembilan.

Supplementary Figure 2. Continuous 417-week longitudinal plots for all model input series: pediatric RSV cases, co-circulating viral antigens (INFV, ADEV, HMPV, PARAV), meteorological variables (mean temperature, total rainfall, relative humidity, wind speed), and weekly public/school holiday days.

Supplementary Figure 3. Weekly RSV case intensity heatmaps across three sociodemographically distinct representative districts in Negeri Sembilan: Seremban (major urban center), Port Dickson (coastal district), and Kuala Pilah (inland district).

Supplementary Figure 4. Diagnostic plots for the final ARIMAX (2,0,4) (1,0,0)_52_ model residuals. (A) Standardized residual time series. (B) Autocorrelation Function (ACF) plot of residuals up to lag 52. (C) Histogram and fitted Gaussian kernel density curve of standardized residuals. (D) p-values for the Ljung-Box portmanteau test evaluated across sequential lags
